# Obesity is not associated with recurrent venous thromboembolism in elderly patients: Results from the prospective SWITCO65+ cohort study

**DOI:** 10.1371/journal.pone.0184868

**Published:** 2017-09-15

**Authors:** Carolin Mueller, Andreas Limacher, Marie Méan, Nicolas Rodondi, Drahomir Aujesky

**Affiliations:** 1 Department of General Internal Medicine, Bern University Hospital, University of Bern, Bern, Switzerland; 2 CTU Bern, Department of Clinical Research, and Institute of Social and Preventive Medicine (ISPM), University of Bern, Bern, Switzerland; 3 Department of General Internal Medicine, Lausanne University Hospital, Lausanne, Switzerland; 4 Institute of Primary Health Care (BIHAM), University of Bern, Bern, Switzerland; Maastricht University Medical Center, NETHERLANDS

## Abstract

**Background:**

Whether obesity is associated with recurrent venous thromboembolism (VTE) in elderly patients is unknown.

**Objectives:**

To examine the association between two obesity measures, the body mass index (BMI) and the waist circumference (WC), and recurrent VTE in elderly patients.

**Patients/Methods:**

We studied 986 patients aged ≥65 years with an acute VTE from a prospective multicenter cohort study (09/2009-12/2013). The BMI was determined and categorized as <25, 25 to <30, or ≥30 kg/m^2^. The WC was categorized as <80 cm in women (w)/<94 cm in men (m), 80 to <88 cm (w)/94 to <102 cm (m), or ≥88 cm (w)/≥102 cm (m). We examined the association between the BMI and the WC and the time to a first symptomatic recurrent VTE using competing risk regression, adjusting for known risk factors of VTE recurrence and periods of anticoagulation.

**Results:**

The mean follow-up was 28 months. The 3-year cumulative incidence of recurrent VTE did not vary by BMI and was 17.6% for a BMI <25 kg/m^2^, 11.5% for a BMI 25 to <30 kg/m^2^, and 16.9% for a BMI ≥30 kg/m^2^ (*P* = 0.09). The 3-year cumulative incidence of recurrent VTE did not vary by WC. After adjustment, neither the BMI (sub-hazard ratio [SHR] 1.02, 95% confidence interval [CI 0.98–1.05]) nor the WC (SHR 1.01, 95% CI 0.99–1.02) was associated with recurrent VTE.

**Conclusions:**

Measures of body weight were not associated with recurrent VTE in our cohort. Obesity does not appear to be a predictor of recurrent VTE in the elderly.

## Introduction

While obesity is an established risk factor for a first venous thromboembolism (VTE) [1–4], whether obesity is also associated with recurrent VTE is controversial. Several studies found that a higher body mass index (BMI) is independently associated with recurrent VTE [5–7] but others did not [8–10].

More than 60% of VTE episodes occur in patients aged 65 years or older [11]. As elderly patients were underrepresented in prior studies demonstrating a relationship between an increased BMI and recurrent VTE (mean patient age 49–62 years) [5–7], whether obesity is a risk factor for recurrent VTE in the elderly is unknown. Moreover, as the muscle mass decreases in older age and is replaced by fat (“sarcopenic obesity”), the BMI may not increase with obesity in the elderly, and the waist circumference (WC), a measure of abdominal fat distribution, may be more important than the BMI to examine health risks associated with obesity in the elderly [12]. We evaluated the association between two measures of body weight, the BMI and the WC, and recurrent VTE in a prospective cohort of elderly patients with acute VTE. We also examined the quality of anticoagulation across BMI categories.

## Patients and methods

The study was conducted between September 2009 and December 2013 as part of the SWIss venous Thromboembolism COhort (SWITCO65+), a prospective multicenter cohort study that assessed long-term medical outcomes in elderly patients with acute VTE from all five university and four high-volume non-university hospitals in Switzerland. Consecutive patients aged ≥65 years with an acute, objectively confirmed symptomatic VTE were identified in the in- and outpatient services of all participating study sites. A detailed description of the study methods was published elsewhere [13]. The Institutional Review Board at each participating center approved the study and patients gave written consent to participation. The approving ethics committee was the “Kantonale Ethikkommission Bern.”

Trained study nurses prospectively collected baseline demographics (age, sex), comorbid conditions (diabetes mellitus, arterial hypertension, immobilization, chronic pulmonary disease, cerebrovascular disease, active cancer, chronic liver and renal disease, heart failure, inflammatory bowel disease, presence of hemiparesis, hemiplegia, or paraplegia, prior varicose vein surgery [as a proxy for varicose veins]), type of the index VTE (unprovoked, provoked, or cancer-related), prior history of VTE, localization of VTE (PE ±DVT vs. DVT alone), family history of DVT or PE, estrogen therapy, concomitant antiplatelet therapy, and VTE-related treatments (low-molecular-weight heparin, unfractionated heparin, fondaparinux, and vitamin K antagonists) using standardized data collection forms. At study entry the BMI was calculated (weight in kilograms divided by height in meters squared) and categorized as normal (<25 kg/m^2^), overweight (25 to <30 kg/m^2^), and obese (≥30 kg/m^2^) [14]. The WC was measured in cm at the umbilical line and categorized as normal (<80 cm in women [w] / <94 cm in men [m]), overweight (80 to <88 cm [w] / 94 to <102 cm [m]), and obese (≥88 cm [w] / ≥102 cm [m]) [14, 15].

The outcome was the time to a first recurrent VTE during follow-up, defined as new or recurrent, symptomatic, and objectively confirmed pulmonary embolism (PE), including fatal PE, or proximal and/or distal deep vein thrombosis (DVT) based on previously published criteria [13]. We defined distal DVT as any DVT below the popliteal vein. Follow-up started after enrolment and included one telephone interview and two surveillance face-to-face evaluations during the first year of study participation, then semi-annual contacts alternating between face-to-face evaluations (clinic visits or home visits in house-bound patients) and telephone calls, as well as periodic reviews of the patient’s hospital chart. During each visit/contact, study nurses interviewed patients to obtain information about recurrent VTE and assessed whether or not the patient had died. We also collected international normalized ratio (INR) values throughout the period of follow-up. If a clinical event had occurred, supplemental information was obtained by reviewing medical charts and interviewing the patient’s primary care physician and/or family members. All outcomes were reviewed and adjudicated by a committee of three blinded clinical experts. Based on the full consensus of this committee, deaths were classified as definitely due to PE (e.g., confirmed by autopsy or following severe PE), possibly due to PE (e.g., sudden death without obvious cause), or due to other causes.

We compared the percentage of time spent within one of three specified INR ranges (<2.0, 2.0–3.0, >3.0) across BMI categories according to the Rosendaal method using the non-parametric Kruskal-Wallis rank test [16]. We calculated the incidence rates of VTE by BMI and WC category and also compared the cumulative incidence of a first recurrent VTE using Kaplan-Meier curves and the log-rank test. We examined the association between obesity measures and the time to a first VTE recurrence using competing risk regression, accounting for non-VTE related death as a competing event [17]. We adjusted for risk factors that have been previously shown to be associated with recurrent VTE, including age, sex, heart failure, inflammatory bowel disease, presence of hemiparesis, hemiplegia, or paraplegia, prior varicose vein surgery (as a proxy for varicose veins), type of the index VTE (unprovoked, provoked, or cancer-related), prior history of VTE, localization of VTE (PE ±DVT vs. DVT alone), family history of DVT or PE, and periods of anticoagulation as a time-varying covariate [6, 18–30]. We repeated the same analyses using percentile-based BMI/WC categories (>10^th^, 10-90^th^, and >90^th^ percentile). We further stratified analyses by sex and age group (65–75 vs. >75 years) and performed several sensitivity analyses by excluding patients with prior VTE, cancer, BMI <18.5 kg/m^2^ and isolated distal DVT. We also excluded the period of initial anticoagulation in another sensitivity analysis. All analyses were done using Stata 14 (Stata Corp., College Station, Texas).

## Results

Of the 1863 screened patients aged ≥65 years with acute VTE, 462 had at least one exclusion criterion and 398 did not consent to participate. Of the 1003 participating patients, 58 withdrew their consent and four were lost to follow-up. These patients were considered in the analysis, i.e. they were censored at the time of withdrawal or loss to follow-up. We excluded 17 patients (8 denied use of data, 4 withdrew from study within one day, and 5 with no BMI reported), our final study sample comprising 986 elderly patients with acute VTE. Excluded patients were older (median age 78 vs. 75 years, *P*<0.001) and more likely to be female (59% vs. 47%, *P*<0.001) than analyzed patients.

Overall, 342 (35%), 402 (41%), and 242 (24%) patients had a BMI <25 kg/m^2^, 25 to <30 kg/m^2^, and ≥30 kg/m^2^, respectively. Patients with a higher BMI were younger, more likely to be female, more likely to have diabetes mellitus and arterial hypertension, and less likely to have active cancer (Table 1). The WC was available in 890 of 986 patients (90%). Of these, 109 (12%) had a WC <80 cm (w) / <94 cm (m), 157 (18%) a WC 80 to <88 cm (w) / 94 to <102 (m) cm, and 624 (70%), a WC ≥88 cm (w) / ≥102 cm (m).

**Table 1:** Patient baseline characteristics.

Characteristic	All (n = 986)	BMI <25 m^2^/kg (n = 342)	BMI ≥25 to <30 m^2^/kg (n = 402)	BMI ≥30 m^2^/kg (n = 242)
	n (%) or median (interquartile range)			
Age, years	75.0 (69.0–81.0)	77.0 (70.0–83.0)	75.0 (69.0–80.0)	73.0 (69.0–78.0)
Female sex	460 (47)	154 (45)	170 (42)	136 (56)
Diabetes mellitus	154 (16)	37 (11)	57 (14)	60 (25)
Arterial hypertension	633 (64)	182 (53)	256 (64)	195 (81)
Immobilization^ǂ^	217 (22)	80 (23)	84 (21)	53 (22)
Chronic pulmonary disease^¶^	135 (14)	51 (15)	53 (13)	31 (13)
Cerebrovascular disease**	91 (9)	33 (10)	34 (8)	24 (10)
Heart failure^††^	115 (12)	41 (12)	37 (9)	37 (15)
Inflammatory bowel disease	32 (3)	15 (4)	11 (3)	6 (2)
Hemiparesia, hemiplegia, or paraplegia	28 (3)	10 (3)	8 (2)	10 (4)
Prior varicose vein surgery	136 (14)	38 (11)	64 (16)	34 (14)
Type of VTE				
Provoked	214 (22)	77 (23)	85 (21)	52 (21)
Unprovoked*	595 (60)	185 (54)	250 (62)	160 (66)
Cancer-related†	177 (18)	80 (23)	67 (17)	30 (12)
Prior VTE	283 (29)	86 (25)	114 (28)	83 (34)
Localization of VTE				
PE±DVT	682 (69)	225 (66)	279 (69)	178 (74)
PE/proximal DVT	906 (92)	314 (92)	364 (91)	228 (94)
Family history of PE/DVT	165 (17)	47 (14)	66 (16)	52 (21)
Estrogen therapy^§^	32 (3)	16 (5)	12 (3)	4 (2)
Concomitant antiplatelet therapy^¥^	319 (32)	104 (30)	131 (33)	84 (35)
Type of initial parenteral AC				
LMWH	464 (47)	170 (50)	185 (46)	109 (45)
Unfractionated heparin	329 (33)	104 (30)	133 (33)	92 (38)
Fondaparinux	158 (16)	50 (15)	76 (19)	32 (13)
Danaparoid	1 (0)	1 (0)	0 (0)	0 (0)
None	34 (3)	17 (5)	8 (2)	9 (4)
Initial VKA therapy	857 (87)	276 (81)	363 (90)	218 (90)

Abbreviations: BMI = body mass index; VTE = venous thromboembolism; PE = pulmonary embolism; DVT = deep vein thrombosis; AC = anticoagulation; LMWH = Low-molecular-weight-heparin; VKA = vitamin K antagonist.

^ǂ^Immobilization (bed rest >72 hours, fracture or cast of the lower extremity, voyage in sitting position >6 hours) during the last 3 months before index VTE.

^¶^Chronic obstructive pulmonary disease, active asthma, lung fibrosis, cystic fibrosis, or bronchiectasis.

**History of ischemic or hemorrhagic stroke or hemiparesis, hemiplegia, or paraplegia at the time of screening.

^††^Known history of systolic or diastolic heart failure, left or right heart failure, forward or backward heart failure, left ventricular ejection fraction of <40%, or acute heart failure (NYHA III/IV) during the last 3 months.

*Any VTE unrelated to cancer or major surgery, immobilization, or estrogen therapy during the last 3 months before index VTE.

^†^Solid or hematologic cancer requiring chemotherapy, radiotherapy, surgery, and/or palliative care during the last 3 month before index VTE.

^§^Estrogen therapy during the last 3 months before index VTE.

^¥^Use of aspirin, clopidogrel, prasugrel, aspirin/dipyramidol.

**Fig 1:**
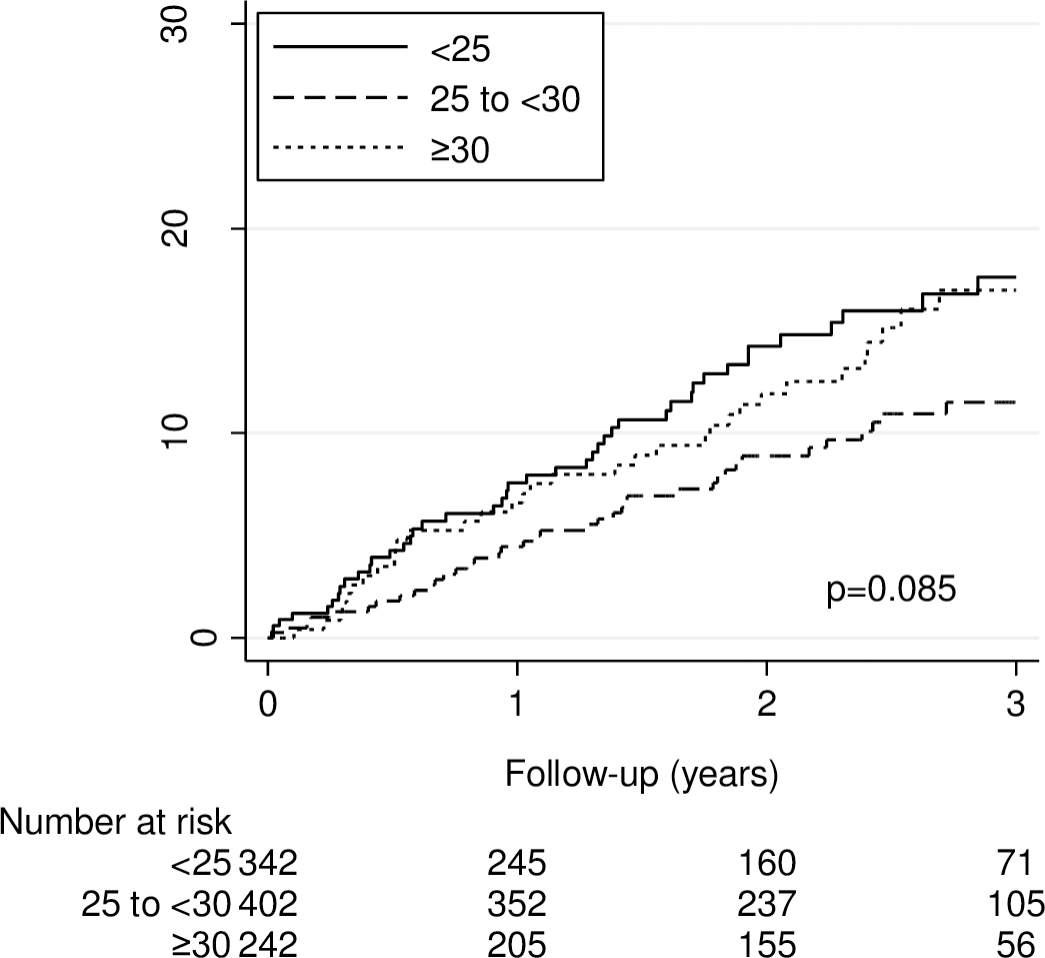
Kaplan-Meier estimates of a first recurrent venous thromboembolism by body mass index (in kg/m^2^). The 3-year cumulative incidence of a first recurrent venous thromboembolism was 17.6%, 11.5%, and 17.0% for patients with a body mass index <25, 25 to <30, and >30 kg/m^2^, respectively (*P* = 0.09 by the logrank test).

**Fig 2:**
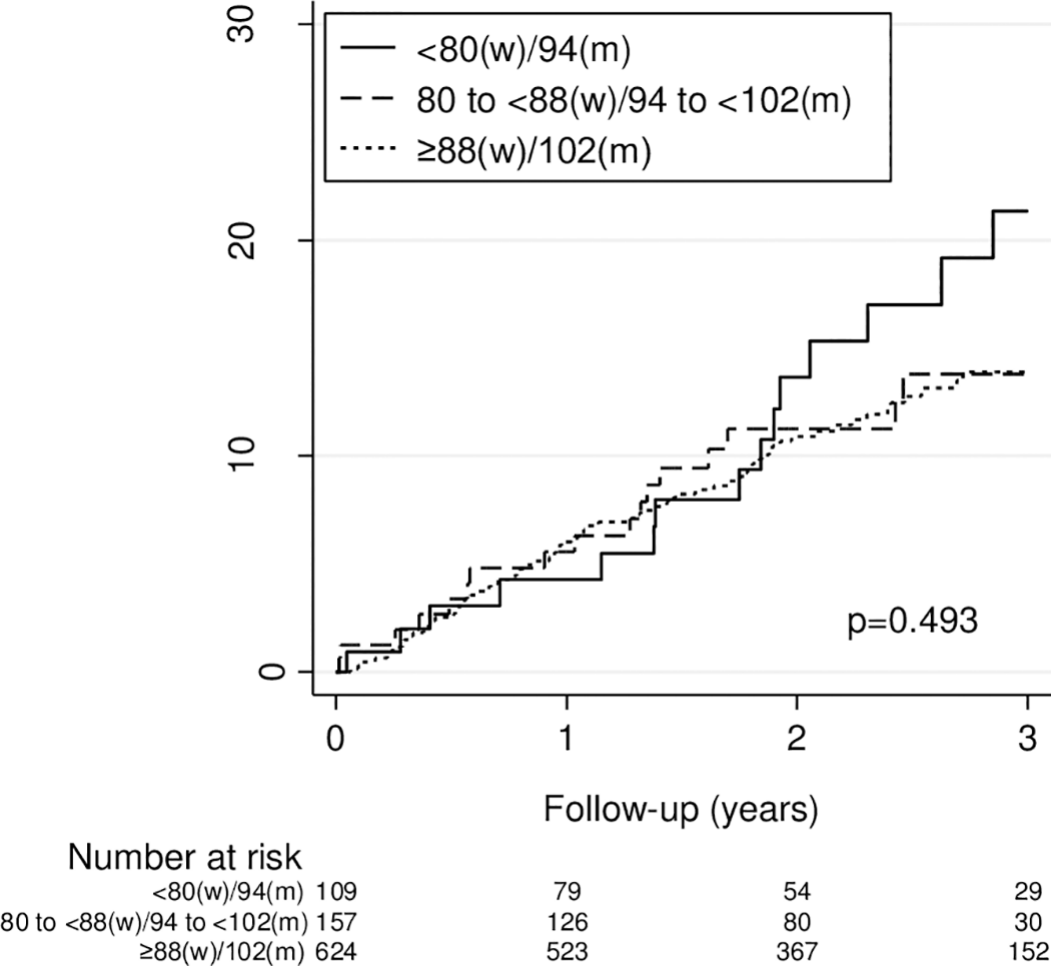
Kaplan-Meier estimates of a first recurrent venous thromboembolism by waist circumference (in cm). The 3-year cumulative incidence of a first recurrent VTE was 21.4%, 13.8%, and 13.9% for patients with a waist circumference <80 (w) / <94 (m), 80 to <88 (w) / 94 to <102 (m), and ≥88 cm (w) / ≥102 cm (m), respectively (*P* = 0.49 by the logrank test).

After a median follow-up period of 30 months (interquartile range [IQR] 19 to 36), 203 (20%) of 986 patients died and 122 (12%) experienced a first recurrent VTE. Of the patients with recurrent VTE, 82 (67%) had PE±DVT and 40 (33%) had DVT only. Overall, 23 of 82 (28%) patients had fatal PE. The median duration of initial anticoagulation was 7.5 months (IQR 4.0 to 24.0). The 3-year cumulative incidence of recurrent VTE did not vary by BMI and was 17.6% for patients with a BMI <25 kg/m^2^, 11.5% for patients with a BMI 25 to <30 kg/m^2^, and 16.9% for patients with a BMI ≥30 kg/m^2^ (*P* = 0.09) (Fig 1). Similarly, the 3-year cumulative incidence of recurrent VTE did not vary by WC and was 21% for patients with a WC <80 cm (w) / <94 cm (m), 14% for patients with a WC 80 to <88 cm (w) / 94 to <102 cm (m), and 14% for patients with a WC ≥88 cm (w) / ≥102 cm (m) (*P* = 0.49) (Fig 2).

Overall, patients spent 65% of their time in the therapeutic INR range (2.0–3.0). The time spent in therapeutic INR range was 62%, 67%, and 64% for patients with a BMI <25 kg/m^2^, 25 to <30 kg/m^2^, and ≥30 kg/m^2^, respectively (*P* = 0.045), with patients with a BMI <25 kg/m^2^ and ≥30 kg/m^2^ spending more time in the subtherapeutic INR range (<2.0) (*P* = 0.012) (Table 2). The time spent in the supratherapeutic INR range (>3.0) did not vary across BMI categories. The median duration of initial anticoagulation was 6.6 (IQR 3.1 to 23.2), 7.5 (IQR 4.9 to 24.0) and 9.4 (IQR 5.0 to 28.4) in patients with a BMI <25 kg/m^2^, 25 to <30 kg/m^2^, and ≥30 kg/m^2^, respectively (*P* = 0.006).

**Table 2:** Percentage of time in a given INR range by body mass index.

INR range*	BMI <25 m^2^/kg (N = 254)	BMI 25 to <30 m^2^/kg (N = 351)	BMI ≥30 m^2^/kg (N = 213)	*P*-value
	Median percentage (interquartile range)			
<2.0	19 (8–38)	13 (5–31)	18 (7.4–33)	0.012
2.0–3.0	62 (46–79)	67 (49–82)	64 (49–78)	0.045
>3.0	10 (3–20)	9 (2.5–20.5)	11 (4–22)	0.292

Abbreviations: INR = international normalized ratio; BMI = body mass index.

*Only patients with initial VKA and at least two INR measurements were considered (n = 818).

After adjustment for known risk factors of recurrent VTE and periods of anticoagulation, patients with a BMI 25 to <30 kg/m^2^ (adjusted sub-hazard ratio [SHR] 0.78, 95% confidence interval [CI] 0.51–1.20) and a BMI ≥30 kg/m^2^ (SHR 1.10, 95% CI 0.70–1.74) did not have higher risk of recurrent VTE compared to patients with a BMI <25 kg/m^2^ (Table 3). There was no association between BMI as a continuous variable and recurrent VTE (SHR 1.02, 95% CI 0.98–1.05). Similarly, we found no association between WC as categorical or continuous variable and recurrent VTE (Table 3). Stratified analyses yielded similar results for men vs. women (S1 Table) and for patients aged 65 to 75 years vs. >75 years (S2 Table). When using percentile-based cut-offs for obesity measures, we did not find a higher incidence rate and risk of recurrent VTE in patients in the 90^th^ BMI and WC percentile (S3 Table). Similarly, when we excluded patients with cancer (S4 Table), isolated distal DVT (S5 Table), BMI <18.5 kg/m^2^ (S6 Table), and prior VTE (S7 Table) in sensitivity analyses, there was no association between excess body weight and VTE recurrence. When we considered only follow up periods after the completion of the initial anticoagulation period in a sensitivity analysis, the results did not change (data not shown).

**Table 3:** Association between obesity measures and recurrent venous thromboembolism.

Measure of obesity	No of events/patients	IR (95% CI)	Adjusted SHR* (95% CI)
**Body mass index, kg/m**^**2**^			
Categorized			
<25	44/342	6.7 (5.0 to 9.0)	Reference
25 to <30	43/402	4.6 (3.4 to 6.3)	0.79 (0.52–1.22)
≥30	35/242	6.3 (4.5 to 8.8)	1.12 (0.71–1.78)
Continuous, per unit	122/986	5.7 (4.8 to 6.8)	1.02 (0.98–1.06)
**Waist circumference, cm**			
Categorized			
<80 (w) / <94 (m)	15/109	6.8 (4.1 to 11.3)	Reference
80 to <88 (w) / 94 to <102 (m)	18/157	5.6 (3.5 to 8.9)	0.89 (0.45–1.78)
≥88 (w) / ≥102 (m)	76/624	5.4 (4.3 to 6.8)	0.98 (0.55–1.72)
Continuous, per unit	109/890	5.6 (4.7 to 6.8)	1.01 (0.99–1.02)

Abbreviations; IR = incidence rate; CI = confidence interval, SHR = sub-hazard ratio.

*Adjusted for age, sex, heart failure, inflammatory bowel disease, presence of hemiparesis, hemiplegia, or paraplegia, prior varicose vein surgery (as a proxy for varicose veins), type of the index VTE (unprovoked, provoked, or cancer-related), prior history of VTE, localization of VTE (PE ±DVT vs. DVT alone), family history of DVT or PE, and periods of anticoagulation as a time-varying covariate

## Discussion

In this prospective multicenter cohort of elderly patients with acute VTE, neither the BMI nor the WC was associated with recurrent VTE. Overall, anticoagulation quality did not substantially vary across BMI categories. In contrast to prior cohort studies enrolling mostly younger patients with VTE (mean age 49–62 years) [5–10], the median age was 75 years in our cohort.

While we found no association between measures of body weight and VTE recurrence, several factors could attenuate a potential relationship between obesity and recurrent VTE in the elderly. As the body weight physiologically decreases after the age of 70 years [31], obese patients were younger and potentially healthier than non-obese patients in our study, as shown by the lower prevalence of cancer in obese patients. Moreover, unknown confounders such as systemic diseases could be responsible for weight loss and cause a higher risk of recurrent VTE in non-obese patients. Thus, we cannot exclude the possibility that despite adjustment for known risk factors such as cancer, patients with a higher body weight may have been healthier and at lower risk of recurrent VTE. Overall, our results indicate that an increased body weight does not confer a higher risk of recurrent VTE in elderly patients and that weight loss is unlikely to result in a reduced thrombosis risk in obese older patients with VTE. The results remained unchanged in various subgroup and sensitivity analyses, which further strengthens our conclusion that obesity is not associated with recurrent VTE in elderly patients.

Our study has potential limitations. First, BMI and WC were only measured at study entry and potential weight changes during follow up could not be considered. Finally, because only 2.4% of patients had a BMI <18.5 kg/m^2^, we could not explore whether underweight, possibly due to comorbid conditions, was associated with recurrent VTE. However, the results did not change when patients with a BMI <18.5 kg/m^2^ were excluded, confirming the robustness of our findings.

In conclusion, measures of body weight were not associated with recurrent VTE in our prospective cohort of elderly patient. Obesity does not appear to be a predictor of recurrent VTE in the elderly and weight loss is unlikely to result in a reduced thrombosis risk in obese older patients with VTE.

## Supporting information

S1 Table Association between obesity measures and recurrent VTE by sex. (DOCX)

S2 Table Association between obesity measures and recurrent VTE by age. (DOCX)

S3 Table Association between obesity measures categorized using percentiles and recurrent VTE. (DOCX)

S4 Table Association between obesity measures and recurrent VTE excluding patients with cancer. (DOCX)

S5 Table Association between obesity measures and recurrent VTE excluding patients with isolated distal DVT. (DOCX)

S6 Table Association between obesity measures and VTE excluding patients with a body mass index <18.5kg/m^2^. (DOCX)

S7 Table Association between obesity measures and recurrent VTE excluding patients with prior VTE. (DOCX)
