# Supplementary material for: Obesity is not associated with recurrent venous thromboembolism in elderly patients: Results from the prospective SWITCO65+ cohort study
Source: PLoS One. 2017 Sep 15;12(9):e0184868. doi: 10.1371/journal.pone.0184868 (PMC5600372; doi:10.1371/journal.pone.0184868)
Supplement: S1 Table — (DOCX) [file pone.0184868.s001.docx]

**S1 Table. Association between obesity measures and recurrent VTE by sex**

| **Measure of obesity** | **No of events/patients** | **IR (95 % CI)** | **Adjusted SHR* (95% CI)** | |
| --- | --- | --- | --- | --- |
| **Female** |  |  |  | |
| **Body mass index, kg/m^2^** |  |  |  | |
| Categorized |  |  |  | |
| <25 | 19/154 | 6.6 (4.2 to 10.3) | Ref. | |
| 25 to <30 | 19/170 | 4.9 (3.1 to 7.7) | 0.85 (0.44 to 1.64) | |
| ≥30 | 19/136 | 6.6 (4.2 to 10.4) | 1.08 (0.52 to 2.24) | |
| Continuous, per unit | 57/460 | 5.9 (4.6 to 7.7) | 1.03 (0.98 to 1.08) |  |
| **Waist circumference, cm** |  |  |  | |
| Categorized |  |  |  | |
| <80 | 1/27 | 2.0 (0.3 to 14.5) | Ref. | |
| 80 to <88 | 6/38 | 7.8 (3.5 to 17.3) | 4.23 (0.56 to 31.96) | |
| ≥88 | 45/354 | 5.9 (4.4 to 7.9) | 3.83 (0.58 to 25.37) | |
| Continuous, per unit | 52/419 | 5.8 (4.4 to 7.6) | 1.01 (0.99 to 1.03) | |
| **Male** |  |  |  | |
| **Body mass index, kg/m^2^** |  |  |  | |
| Categorized |  |  |  | |
| <25 | 25/188 | 6.8 (4.6 to 10.1) | Ref. | |
| 25 to <30 | 24/232 | 4.5 (3.0 to 6.6) | 0.70 (0.38 to 1.26) | |
| ≥30 | 16/106 | 6.0 (3.7 to 9.8) | 1.10 (0.59 to 2.05) | |
| Continuous, per unit | 65/526 | 5.5 (4.3 to 7.1) | 0.99 (0.93 to 1.06) | |
| **Waist circumference, cm** |  |  |  | |
| Categorized |  |  |  | |
| <94 | 14/82 | 8.2 (4.8 to 13.8) | Ref. | |
| 94 to <102) | 12/119 | 4.9 (2.8 to 8.6) | 0.63 (0.29 to 1.41) | |
| ≥102 | 31/270 | 4.9 (3.4 to 7.0) | 0.78 (0.41 to 1.50) | |
| Continuous, per unit | 57/471 | 5.4 (4.2 to 7.0) | 1.00 (0.97 to 1.02) | |

Abbreviations: IR= incidence rate; CI= confidence interval; SHR= sub-hazard ratio.

*Adjusted for age, sex, heart failure, inflammatory bowel disease, presence of hemiparesis, hemiplegia, or paraplegia, prior varicose vein surgery (as a proxy for varicose veins), type of the index VTE (unprovoked, provoked, or cancer-related), prior history of VTE, localization of VTE (PE ±DVT vs. DVT alone), family history of DVT or PE, and periods of anticoagulation as a time-varying covariate.
