# Supplementary material for: Obesity is not associated with recurrent venous thromboembolism in elderly patients: Results from the prospective SWITCO65+ cohort study
Source: PLoS One. 2017 Sep 15;12(9):e0184868. doi: 10.1371/journal.pone.0184868 (PMC5600372; doi:10.1371/journal.pone.0184868)
Supplement: S2 Table — (DOCX) [file pone.0184868.s002.docx]

**S2 Table. Association between obesity measures and VTE by age**

| **Measure of obesity** | **No of events/patients** | **IR (95 % CI)** | **Adjusted SHR* (95% CI)** |
| --- | --- | --- | --- |
| **Age 65-75 years** |  |  |  |
| **Body mass index, kg/m^2^** |  |  |  |
| Categorized |  |  |  |
| <25 | 18/156 | 5.5 (3.5 to 8.7) | Ref. |
| 25 to <30 | 25/218 | 4.9 (3.3 to 7.3) | 0.94 (0.49 to 1.79) |
| ≥30 | 23/149 | 6.6 (4.4 to 10.0) | 1.25 (0.65 to 2.39) |
| Continuous, per unit | 66/523 | 5.6 (4.4 to 7.1) | 1.03 (0.98 to 1.08) |
| **Waist circumference, cm** |  |  |  |
| Categorized |  |  |  |
| <80 (w) / <94 (m) | 11/63 | 8.0 (4.4 to 14.5) | Ref. |
| 80 to <88 (w) / 94 to <102 (m) | 7/81 | 3.9 (1.8 to 8.1) | 0.53 (0.20 to 1.36) |
| ≥88 (w) / ≥102 (m) | 41/329 | 5.4 (4.0 to 7.4) | 0.75 (0.37 to 1.50) |
| Continuous, per unit | 59/473 | 5.5 (4.3 to 7.1) | 1.01 (0.99 to 1.03) |
| **Age >75 years** |  |  |  |
| **Body mass index, kg/m^2^** |  |  |  |
| Categorized |  |  |  |
| <25 | 26/186 | 7.9 (5.4 to 11.6) | Ref. |
| 25 to <30 | 18/184 | 4.3 (2.7 to 6.8) | 0.68 (0.36 to 1.28) |
| ≥30 | 12/93 | 5.8 (3.3 to 10.2) | 0.96 (0.45 to 2.01) |
| Continuous, per unit | 56/463 | 5.9 (4.5 to 7.6) | 1.00 (0.94 to 1.05) |
| **Waist circumference, cm** |  |  |  |
| Categorized |  |  |  |
| <80 (w) / <94 (m) | 4/46 | 4.9 (1.8 to 12.9) | Ref. |
| 80 to <88 (w) / 94 to <102 (m) | 11/76 | 7.8 (4.3 to 14.2) | 1.91 (0.58 to 6.28) |
| ≥88 (w) / ≥102 (m) | 35/295 | 5.4 (3.9 to 7.5) | 1.60 (0.53 to 4.88) |
| Continuous, per unit | 50/417 | 5.8 (4.4 to 7.6) | 1.00 (0.98 to 1.03) |

Abbreviations: IR= incidence rate; CI= confidence interval; SHR= sub-hazard ratio.

*Adjusted for age, sex, heart failure, inflammatory bowel disease, presence of hemiparesis, hemiplegia, or paraplegia, prior varicose vein surgery (as a proxy for varicose veins), type of the index VTE (unprovoked, provoked, or cancer-related), prior history of VTE, localization of VTE (PE ±DVT vs. DVT alone), family history of DVT or PE, and periods of anticoagulation as a time-varying covariate.
