# Supplementary material for: Obesity is not associated with recurrent venous thromboembolism in elderly patients: Results from the prospective SWITCO65+ cohort study
Source: PLoS One. 2017 Sep 15;12(9):e0184868. doi: 10.1371/journal.pone.0184868 (PMC5600372; doi:10.1371/journal.pone.0184868)
Supplement: S3 Table — (DOCX) [file pone.0184868.s003.docx]

**S3 Table. Association between obesity measures categorized using percentiles and recurrent VTE**

| **Measure of obesity** | **No of events/patients** | **IR (95 % CI)** | **Adjusted SHR* (95% CI)** |
| --- | --- | --- | --- |
| **Body mass index (percentiles)** |  |  |  |
| 10 to 90th percentile | 99/793 | 5.6 (4.6 to 6.9) | Ref. |
| <10th percentile | 11/96 | 6.6 (3.7 to 12.0) | 0.99 (0.51 to 1.93) |
| >90th percentile | 12/97 | 5.5 (3.1 to 9.8) | 1.02 (0.55 to 1.90) |
| **Waist circumference (percentiles)** |  |  |  |
| 10 to 90th percentile | 82/713 | 5.2 (4.2 to 6.5) | Ref. |
| <10th percentile | 12/89 | 6.6 (3.8 to 11.6) | 1.04 (0.53 to 2.01) |
| >90th percentile | 15/88 | 7.8 (4.7 to 12.9) | 1.58 (0.88 to 2.82) |

Abbreviations: IR= incidence rate; CI= confidence interval; SHR= sub-hazard ratio.

*Adjusted for age, sex, heart failure, inflammatory bowel disease, presence of hemiparesis, hemiplegia, or paraplegia, prior varicose vein surgery (as a proxy for varicose veins), type of the index VTE (unprovoked, provoked, or cancer-related), prior history of VTE, localization of VTE (PE ±DVT vs. DVT alone), family history of DVT or PE, and periods of anticoagulation as a time-varying covariate.
