# Supplementary material for: Obesity is not associated with recurrent venous thromboembolism in elderly patients: Results from the prospective SWITCO65+ cohort study
Source: PLoS One. 2017 Sep 15;12(9):e0184868. doi: 10.1371/journal.pone.0184868 (PMC5600372; doi:10.1371/journal.pone.0184868)
Supplement: S4 Table — (DOCX) [file pone.0184868.s004.docx]

**S4 Table. Association between obesity measures and recurrent VTE excluding patients with cancer**

| **Measure of obesity** | **No of events/patients** | **IR (95 % CI)** | **Adjusted SHR* (95% CI)** |
| --- | --- | --- | --- |
| **Body mass index, kg/m^2^** |  |  |  |
| Categorized |  |  |  |
| <25 | 37/262 | 6.4 (4.7 to 8.9) | Ref. |
| 25 to <30 | 37/335 | 4.6 (3.3 to 6.3) | 0.76 (0.48 to 1.21) |
| ≥30 | 31/212 | 6.3 (4.4 to 8.9) | 1.09 (0.67 to 1.78) |
| Continuous, per unit | 105/809 | 5.6 (4.6 to 6.8) | 1.02 (0.98 to 1.06) |
| **Waist circumference, cm** |  |  |  |
| Categorized |  |  |  |
| <80 (w) / <94 (m) | 11/86 | 5.6 (3.1 to 10.1) | Ref. |
| 80 to <88 (w) / 94 to <102 (m) | 16/113 | 6.3 (3.8 to 10.2) | 1.12 (0.52 to 2.41) |
| ≥88 (w) / ≥102 (m) | 66/534 | 5.3 (4.1 to 6.7) | 1.04 (0.55 to 1.99) |
| Continuous, per unit | 93/733 | 5.5 (4.5 to 6.7) | 1.01 (0.99 to 1.02) |

Abbreviations: IR= incidence rate; CI= confidence interval; SHR= sub-hazard ratio.

*Adjusted for age, sex, heart failure, inflammatory bowel disease, presence of hemiparesis, hemiplegia, or paraplegia, prior varicose vein surgery (as a proxy for varicose veins), type of the index VTE (unprovoked, provoked, or cancer-related), prior history of VTE, localization of VTE (PE ±DVT vs. DVT alone), family history of DVT or PE, and periods of anticoagulation as a time-varying covariate.
