# Supplementary material for: Obesity is not associated with recurrent venous thromboembolism in elderly patients: Results from the prospective SWITCO65+ cohort study
Source: PLoS One. 2017 Sep 15;12(9):e0184868. doi: 10.1371/journal.pone.0184868 (PMC5600372; doi:10.1371/journal.pone.0184868)
Supplement: S5 Table — (DOCX) [file pone.0184868.s005.docx]

**S5 Table. Association between obesity measures and recurrent VTE excluding patients with isolated distal DVT**

| **Measure of obesity** | **No of events/patients** | **IR (95 % CI)** | **Adjusted SHR* (95% CI)** |
| --- | --- | --- | --- |
| **Body mass index, kg/m^2^** |  |  |  |
| Categorized |  |  |  |
| <25 | 41/314 | 6.8 (5.0 to 9.3) | Ref. |
| 25 to <30 | 40/364 | 4.8 (3.5 to 6.5) | 0.80 (0.51 to 1.25) |
| ≥30 | 33/228 | 6.4 (4.5 to 9.0) | 1.11 (0.69 to 1.80) |
| Continuous, per unit | 114/906 | 5.8 (4.9 to 7.0) | 1.02 (0.98 to 1.06) |
| **Waist circumference, cm** |  |  |  |
| Categorized |  |  |  |
| <80 (w) / <94 (m) | 14/98 | 7.0 (4.2 to 11.9) | Ref. |
| 80 to <88 (w) / 94 to <102 (m) | 17/141 | 6.0 (3.7 to 9.6) | 0.98 (0.48 to 2.02) |
| ≥88 (w) / ≥102 (m) | 72/574 | 5.6 (4.5 to 7.1) | 0.99 (0.56 to 1.77) |
| Continuous, per unit | 103/813 | 5.8 (4.8 to 7.1) | 1.01 (0.99 to 1.02) |

Abbreviations: IR= incidence rate; CI= confidence interval; SHR= sub-hazard ratio.

*Adjusted for age, sex, heart failure, inflammatory bowel disease, presence of hemiparesis, hemiplegia, or paraplegia, prior varicose vein surgery (as a proxy for varicose veins), type of the index VTE (unprovoked, provoked, or cancer-related), prior history of VTE, localization of VTE (PE ±DVT vs. DVT alone), family history of DVT or PE, and periods of anticoagulation as a time-varying covariate.
