# Supplementary material for: Obesity is not associated with recurrent venous thromboembolism in elderly patients: Results from the prospective SWITCO65+ cohort study
Source: PLoS One. 2017 Sep 15;12(9):e0184868. doi: 10.1371/journal.pone.0184868 (PMC5600372; doi:10.1371/journal.pone.0184868)
Supplement: S6 Table — (DOCX) [file pone.0184868.s006.docx]

**S6 Table. Association between obesity measures and recurrent VTE excluding patients with a body mass index <18.5 kg/m^2^**

| **Measure of obesity** | **No of events/patients** | **IR (95 % CI)** | **Adjusted SHR* (95% CI)** |
| --- | --- | --- | --- |
| **Body mass index, kg/m^2^** |  |  |  |
| Categorized |  |  |  |
| <25 | 42/318 | 6.8 (5.0 to 9.1) | Ref. |
| 25 to <30 | 43/402 | 4.6 (3.4 to 6.3) | 0.77 (0.50 to 1.20) |
| ≥30 | 35/242 | 6.3 (4.5 to 8.8) | 1.09 (0.68 to 1.73) |
| Continuous, per unit | 120/962 | 5.7 (4.8 to 6.8) | 1.02 (0.98 to 1.06) |
| **Waist circumference, cm** |  |  |  |
| Categorized |  |  |  |
| <80 (w) / <94 (m) | 14/98 | 6.8 (4.0 to 11.5) | Ref. |
| 80 to <88 (w) / 94 to <102 (m) | 17/148 | 5.5 (3.4 to 8.9) | 0.89 (0.43 to 1.82) |
| ≥88 (w) / ≥102 (m) | 76/621 | 5.4 (4.4 to 6.8) | 0.95 (0.53 to 1.71) |
| Continuous, per unit | 107/867 | 5.6 (4.6 to 6.8) | 1.00 (0.99 to 1.02) |

Abbreviations: IR= incidence rate; CI= confidence interval; SHR= sub-hazard ratio.

*Adjusted for age, sex, heart failure, inflammatory bowel disease, presence of hemiparesis, hemiplegia, or paraplegia, prior varicose vein surgery (as a proxy for varicose veins), type of the index VTE (unprovoked, provoked, or cancer-related), prior history of VTE, localization of VTE (PE ±DVT vs. DVT alone), family history of DVT or PE, and periods of anticoagulation as a time-varying covariate.
