# Supplementary material for: Obesity is not associated with recurrent venous thromboembolism in elderly patients: Results from the prospective SWITCO65+ cohort study
Source: PLoS One. 2017 Sep 15;12(9):e0184868. doi: 10.1371/journal.pone.0184868 (PMC5600372; doi:10.1371/journal.pone.0184868)
Supplement: S7 Table — (DOCX) [file pone.0184868.s007.docx]

**S7 Table. Association between obesity measures and recurrent VTE excluding patients with prior VTE**

| **Measure of obesity** | **No of events/patients** | **IR (95 % CI)** | **Adjusted SHR* (95% CI)** |
| --- | --- | --- | --- |
| **Body mass index, kg/m^2^** |  |  |  |
| Categorized |  |  |  |
| <25 | 31/256 | 6.4 (4.5 to 9.0) | Ref. |
| 25 to <30 | 34/288 | 5.2 (3.7 to 7.3) | 0.94 (0.58 to 1.54) |
| ≥30 | 24/159 | 6.6 (4.4 to 9.8) | 1.23 (0.72 to 2.12) |
| Continuous, per unit | 89/703 | 5.9 (4.8 to 7.3) | 1.03 (0.99 to 1.07) |
| **Waist circumference, cm** |  |  |  |
| Categorized |  |  |  |
| <80 (w) / <94 (m) | 10/79 | 6.3 (3.4 to 11.6) | Ref. |
| 80 to <88 (w) / 94 to <102 (m) | 15/121 | 6.3 (3.8 to 10.4) | 1.11 (0.50 to 2.47) |
| ≥88 (w) / ≥102 (m) | 55/445 | 5.6 (4.3 to 7.3) | 1.08 (0.54 to 2.13) |
| Continuous, per unit | 80/645 | 5.8 (4.6 to 7.2) | 1.01 (0.99 to 1.02) |

Abbreviations: IR= incidence rate; CI= confidence interval; SHR= sub-hazard ratio.

*Adjusted for age, sex, heart failure, inflammatory bowel disease, presence of hemiparesis, hemiplegia, or paraplegia, prior varicose vein surgery (as a proxy for varicose veins), type of the index VTE (unprovoked, provoked, or cancer-related), prior history of VTE, localization of VTE (PE ±DVT vs. DVT alone), family history of DVT or PE, and periods of anticoagulation as a time-varying covariate.
